# Uncertainty in identifying local extinctions: the distribution of missing data and its effects on biodiversity measures

**DOI:** 10.1098/rsbl.2015.0824

**Published:** 2016-03

**Authors:** Elizabeth H. Boakes, Richard A. Fuller, Philip J. K. McGowan, Georgina M. Mace

**Affiliations:** 1 Centre for Biodiversity and Environment Research, University College London, Gower Street, London WC1E 6BT, UK; 2 School of Biological Sciences, University of Queensland, Brisbane, Queensland 4072, Australia; 3 School of Biology, Newcastle University, Newcastle upon Tyne NE1 7RU, UK

**Keywords:** biodiversity monitoring, extinction inference, galliformes, local extinction, spatial bias, species occurrence data

## Abstract

Identifying local extinctions is integral to estimating species richness and geographic range changes and informing extinction risk assessments. However, the species occurrence records underpinning these estimates are frequently compromised by a lack of recorded species absences making it impossible to distinguish between local extinction and lack of survey effort—for a rigorously compiled database of European and Asian Galliformes, approximately 40% of half-degree cells contain records from before but not after 1980. We investigate the distribution of these cells, finding differences between the Palaearctic (forests, low mean human influence index (HII), outside protected areas (PAs)) and Indo-Malaya (grassland, high mean HII, outside PAs). Such cells also occur more in less peaceful countries. We show that different interpretations of these cells can lead to large over/under-estimations of species richness and extent of occurrences, potentially misleading prioritization and extinction risk assessment schemes. To avoid mistakes, local extinctions inferred from sightings records need to account for the history of survey effort in a locality.

## 1. Introduction

Identifying local extinctions is central to documenting changing geographic ranges and informing assessments of species extinction risk. However, species records are frequently collected opportunistically, and so tend to be presence-only, i.e. recorders report what they see but do not record what they did not see/where they did not survey. It is then impossible to establish if a species is present but not recorded, or genuinely absent.

Local extinction can be inferred using a time-series of sightings, providing the area has experienced some continuing survey effort [1]. However, survey effort is often heavily biased in time and space [2] and, in the past 40 years or so, biodiversity records have become increasingly focused on areas of high biodiversity, conservation value and protection [3]. In the absence of any information on survey effort, assumptions have to be made about data-absences, either that no local extinctions have occurred, or that all recent data-absences reflect local extinction. Alternative assumptions can use records of other species to estimate the survey effort. These assumptions have potentially significant impacts on biodiversity metrics such as species richness or range area.

Survey effort may vary predictably. For example, it might be lower in areas where there are few national resources for monitoring, high levels of warfare/political instability, low human influence (e.g. low human population density, lack of transport infrastructure) and low levels of biodiversity. It might vary with vegetation type, with some biomes being easier to survey and more commonly visited. On the other hand, destruction of natural vegetation or areas of high human influence might be an indication of true local extinction.

Here, we test these predictions using a near-exhaustively compiled database of historical and contemporary location records of species in the avian order Galliformes [3]. We (i) explore the distribution of missing data in relation to geographical, ecological and socio-political factors and (ii) investigate the effect that the uncertainty over local extinction has on estimates of species richness and geographic range size calculated under four alternative assumptions about missing presence/absence information.

## 2. Material and methods

### (a) Species occurrence and distribution data

Species occurrence data were collected for the 126 species of Galliformes found in the Palaearctic and Indo-Malaya ([3,4]; electronic supplementary material, S3). The database contained 153 150 records, dating from 1727 to 2008, although records increase markedly through time (electronic supplementary material, S4). Records of species sightings at a point locality (there is no non-sighting information) were included only if they could be accurately dated to within ±10 years, or if the record was known with confidence to have been made before or after 1980. 1980 was chosen as it represents a period of rapid change in many anthropogenic processes [5] and provides a good sample of before and after observations. We aggregated the point locality data into a Behrmann equal area projection, using a grid with cells measuring 48.24 × 48.24 km (approx. half-degree resolution). Grid cell size was chosen to maximize spatial resolution within the constraints of the spatial accuracy of our data, which was approximately half-degree.

### (b) Spatial distribution of data-absent cells

We defined a ‘data-absent’ cell as one that contained at least one record of one species pre-1980, but no records of any species after 1 January 1980, and we studied their distribution at two spatial scales: local- and country-level.

Local-level processes were explored using half-degree cells. We hypothesized that the occurrence of data-absent cells would be affected by (i) biogeographic realm (via a differing history of anthropogenic land conversion and scientific infrastructure); (ii) land cover type (via ease of access for both habitat conversion and conducting surveys); (iii) protected area (PA) status (local extinctions may be more likely to occur outside PAs, PAs may be more attractive to recorders owing to high biodiversity and greater accessibility) and (iv) mean human influence index (HII) [6] per cell (areas of high HII are likely to be both more accessible and more closely associated with local extinction). Cells were allocated to the biogeographic realm, country and land cover type (forest, grassland/shrubland and anthrome, as estimated for 1970, by the HYDE 2.0 model [7]) in which their centroid fell, meaning some coastal cells were excluded. A cell was designated as being within a PA if any part overlapped a PA [8].

We used a binomial generalized linear model with post-1980-data-absence as the binomial response and land cover type (categorical), PA coverage (binomial) and mean HII (continuous) as the explanatory variables (electronic supplementary material, S1). Owing to their different histories of anthropogenic transformation [9], we did not expect the same model to fit the Palaearctic and Indo-Malaya, thus we performed individual analyses for each realm. All statistical analyses were performed in R [10].

At the country level, we hypothesized that data-absent cells would be more likely to occur in countries with fewer financial resources, greater levels of violence/political instability and with an official language that was not English (in collating the data, we might have missed foreign language literature). We performed a generalized linear model on the proportion of data-absent cells per country relative to total cells surveyed against the log of gross domestic product (GDP) *per capita* for 2008 [11,12], the global peace index (GPI) (compiled from 23 indicators such as homicide rates, UN peacekeeping funding) [13], and the binary variable of English as an official language [14] (electronic supplementary material, S2). Covariates were checked for collinearity. We took an information-theoretic approach, ranking possible models by AIC_c_ values using R's MuMIn package [15]. Models that were within two AIC_c_ units of the top ranked model were examined but were not interpreted as being truly competitive if they differed from the best model by one parameter and had essentially the same values of the maximized log-likelihood as the best model [16].

### (c) The effect of uncertainty on biodiversity metrics

We estimated the two biodiversity metrics, (i) species richness (no. species per cell) and (ii) species geographic range size (via extent of occurrence (EOO), calculated in ArcGIS v. 10.0 using a convex hull) for the post-1980 period. We chose EOO as a measure of range, because it should be more robust than area of occupancy to alternative interpretations of data-absence.

These two biodiversity metrics were compared using four different assumptions about the status of species in data-absent cells. Assume

(i) all species that were recorded historically remain extant, i.e. there has been no local extinction, and data-absence is owing to lack of recording effort.
(ii) the likelihood of the species remaining extant within each cell can be inferred from the prior pattern of observations (see electronic supplementary material, S5). Unlike almost all published sighting-rate models [1], our method allows survey effort to fall to zero at any period in the time-series. Sightings occur in a Poisson process with a rate depending on both species presence and survey effort, enabling a resighting probability to be calculated that is used with a threshold of 0.5.
(iii) the species is locally extinct if there is no record of it after 1980 but at least one other species has been recorded in the cell in this time period.
(iv) a species is locally extinct unless it has been recorded post-1980.

## 3. Results

**Figure 1.**
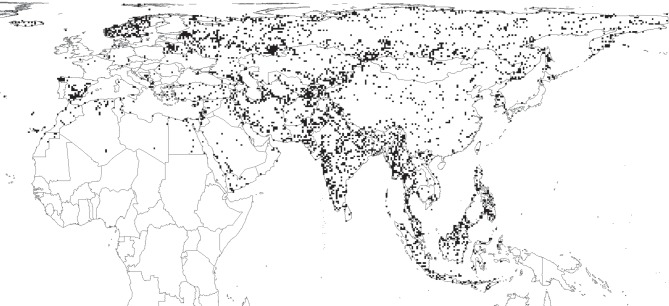
The distribution of data-absent cells, i.e. cells containing at least one record from before 1 January 1980, but no records after this time.

In total, 8672 cells had at least one record from any point in time. Of these cells, almost 40% (3493) were ‘data-absent’ cells, i.e. contained records before but not after 01 January 1980 (figure 1).

### (a) Spatial distribution of data-absent cells

In the Palaearctic, data-absent cells, i.e. cells with records dating from before but not after 1980, were significantly more likely to occur outside PAs, in anthromes and grasslands as opposed to forests and in areas of lower mean HII (electronic supplementary material, S6). In Indo-Malaya, data-absent cells were also significantly more likely to be found outside PAs but in contrast to the Palaearctic, data-absent cells were more likely to be found in areas of high mean HII and in grassland (electronic supplementary material, S6).

The lowest AIC_c_ model contained one predictor, GPI rank, with more peaceful countries having proportionately fewer data-absent cells (*β* = 0.305 ± 0.066; electronic supplementary material, S7). GPI rank was also included in the two models that were within two AIC_c_ units of the lowest AIC_c_ model (electronic supplementary material, S7). These models give weak support to the hypotheses that the percentage of data-absent cells within a country decreases as GDP increases and is lower for countries with an official language that is not English. However, we did not interpret these models as competing with the lowest AIC_c_ model, because the addition of one parameter did not make a difference to the log-likelihood [16].

### (b) Species richness

Species richness per cell differed markedly depending on the assumption made about local extinction (electronic supplementary material, S8). For example, the number of cells with five or more species present post-1980 (approx. the 10% most species-rich cells) under each assumption is as follows (i) 1153; (ii) 833; (iii) 682 and (iv) 631. Such species richness counts are particularly strongly affected in the Himalayas, central India and Southeast Asia (electronic supplementary material, S9).

### (c) Geographic range size

**Figure 2.**
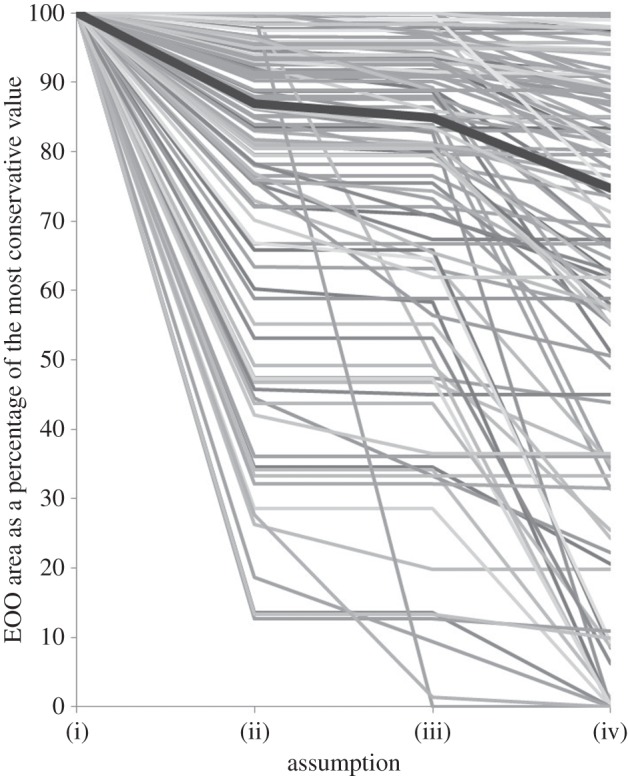
The size of the area of each species' EOO under each assumption as a percentage of its most conservative value (assumption (i)). The thick black line shows the median values.

While 30 species' distributions were sufficiently evenly sampled for the most pessimistic assumption (iv) of their geographical range size to be more than 90% of the most optimistic assumption (i), the EOO estimates were under half the size of their upper limit for 21 species in assumption (ii) (electronic supplementary material, S10); 23 species in assumption (iii) and 28 species in assumption (iv) (figure 2). The EOO estimates were particularly affected in central India, Southeast Asia and the eastern Palaearctic.

## 4. Discussion

Our first analysis examined factors associated with high frequencies of data-absent cells in our database and showed that their distribution differs between the Palaearctic and Indo-Malaya. By 1700, land in Europe was mostly transformed, whereas Asia was only just beginning to undergo conversion that intensified in the twentieth century [9]. The first wave of Palaearctic local extinctions thus occurred much earlier, whereas our analysis should have captured the Indo-Malayan events. The association of data-absence with low mean HII in the Palaearctic may therefore be explained by low survey effort and the association with high mean HII in Indo-Malaya by local extinctions. More difficult to explain is the effect of land cover on data-absence. In the Palaearctic, data-absence was associated with forest but in Indo-Malaya, with grassland. Forests, as the least accessible vegetation, may be more likely to experience low survey effort, whereas data-absence in grasslands, a far greater proportion of which experienced conversion [17], is more likely to be owing to local extinction. However, following this logic, we would expect a high number of local extinctions to occur in Indo-Malayan anthromes, of which we found no evidence. Data-absent cells were more likely to occur outside PAs in both realms, presumably, because (i) PAs should be preventing local extinctions and (ii) scientists and eco-tourists are more likely to visit PAs owing to their greater abundance of biodiversity and accessibility.

At the country-scale, higher proportions of data-absent cells occurred in less peaceful countries, perhaps owing to lower survey effort. Although GDP *per capita* and English-as-an-official-language were not in the best-ranked model, including them as covariates did not increase the model's AIC_c_ substantially and thus, there is some weak support for them as predictors. Lower GDP *per capita* was associated with data-absence, perhaps owing to lower scientific resources that could lead to both lower survey efforts and conservation outcomes. Countries with English as an official language had a lower percentage of data-absent cells, and it is possible that we missed records because of language constraints.

Our analysis showed that different assumptions about data-absent cells can strongly affect estimates of local species richness and geographic range size. In the biodiversity-rich areas of the Himalayas and Southeast Asia, species counts per cell differed by up to 17 species (100%) depending on how the data-absences were treated, compromising the designation of local richness hotspots. EOO estimates were particularly affected by data-absent cells in central India, Southeast Asia and the eastern Palaearctic. Using time-series data to infer extinction (assumption (ii)) yielded approximately 28% fewer species-rich cells than assuming no local extinction (assumption (i)) but nearly 25% more species-rich cells compared with relying on recent data alone (assumption (iv)) and approximately 18% more than assumption (iii), thus in the absence of more complete data seems a sensible compromise. The difference between assumptions (ii) and (iii) with respect to EOO was far less pronounced, with a mean difference in area of only 4%, suggesting that for this measure, at least, a very simple extinction inference model such as assumption (ii) may suffice. However, an understanding of the history of survey effort in an area (as in assumptions (ii) and (iii)) is required for species data to be interpreted for conservation planning.

If the current spatial bias in biodiversity monitoring is not resolved, then inferring future extinctions will become even more problematic in the absence of a spatially representative present-day biodiversity baseline. If monitoring efforts were to be expanded, then one sensible priority would be in areas with accessible historical data.

Overall, our analyses show that the assumptions used to infer local extinction can have a large impact on estimates of species richness and geographic range change. Ultimately, it is critical to ensure that survey effort is accounted for, and that any uncertainties are transparently represented.

## Supplementary Material

BoakesEtAlBiolLettersSupplMaterial1

## Supplementary Material

BoakesEtAlBiolLettersSupplMaterial2

## Supplementary Material

BoakesEtAlBiolLettersSupplMaterial3-10

## Data accessibility

The datasets supporting this article have been uploaded as part of the electronic supplementary material.
