# Supplementary material for: Uncertainty in identifying local extinctions: the distribution of missing data and its effects on biodiversity measures
Source: Biol Lett. 2016 Mar;12(3):20150824. doi: 10.1098/rsbl.2015.0824 (PMC4843216; doi:10.1098/rsbl.2015.0824)

Supplementary Material 3. Data collection methods.

Records were collected from museums (121 of 338 contacted responded), both peer-reviewed and grey literature (the latter from an exhaustive search of the libraries of BirdLife, the Edward Grey Institute, the Institute of Zoology and the World Pheasant Association), bird atlases (17 fitted our data requirements), banding records and two birding trip report websites ([www.birdtours.co.uk](http://www.birdtours.co.uk) and www.travellingbirder.com). Where possible, data were informally refereed by local experts who, if necessary, supplemented the data with their personal records. The research team included volunteers fluent in Chinese, English, French, German, Spanish and Swedish but the vast majority of our data sources were written in English. Whilst the data collection process was as rigorous as it could have been within the constraints of finance (~£8500 spent on data collection), time (~1460 person days) and a UK-base, there will of course be sources that, despite our best efforts, we were unaware of, most likely from locally produced grey literature. It would thus not be unreasonable to expect that records are biased towards English-speaking countries.

Supplementary Material 4. The cumulative number of records over time.

Supplementary Material 5. Modelling a species’ resighting probability.

We assumed that sightings of a particular species in a particular spatial region occur in a Poisson process with rate $\lambda$*(t)* = $\lambda$*e(t)* where *e(t)* reflects the balance between sampling effort and species abundance and is assumed known and $\lambda$ is a constant (unknown) that will vary with region and species. Time varies continuously and we assume a sampling window (0,*T*]. The unknown $\lambda$can be estimated by $\hat{\lambda}$= *N(T)/I(T)* where *N(T)* is the total number of sightings observed in the sampling window, and *I(T)* the total effort, $\int_{0}^{T} e\left( u \right)du$. The probability that there are no sightings in an interval ($\tau,T]$ can be estimated as exp where *I(T)-I($\tau$)* is the total effort over ($\tau,T]$. In discrete time, the probability *p* that there will be no sightings in the years $\tau$+1, ..., *T* is approximated by 1- $\hat{\lambda}$*e(t)*). Effort was measured by the number of years in which at least one sighting of any galliform species (including introduced species) was recorded.

Species were then scored as being extant within a cell in the 1980-2008 time period if *p* ≥ 0.5 (i.e. there is a greater than 50% probability of the species remaining unobserved) or if the species was sighted post 1980. Species were scored as being extinct by 1980 within a cell if the species was not sighted post 1980 and *p* < 0.5.

A worked example is shown here:

|  | 1909 | 1934 | 1940 | 1941 | 1942 | 1946 | 1947 | 1965 | 1969 | 1986 | 1988 | 1994 |
| --- | --- | --- | --- | --- | --- | --- | --- | --- | --- | --- | --- | --- |
| King  quail | 0 | 0 | 0 | 1 | 0 | 0 | 1 | 0 | 1 | 0 | 0 | 0 |
| Grey  junglefowl | 0 | 1 | 0 | 0 | 0 | 0 | 1 | 0 | 1 | 0 | 0 | 1 |
| Green  peafowl | 2 | 0 | 2 | 0 | 1 | 1 | 1 | 4 | 1 | 0 | 1 | 0 |
| Red  junglefowl | 0 | 0 | 0 | 0 | 0 | 0 | 2 | 0 | 1 | 2 | 0 | 0 |

The table shows the number of records per species per year. It should be noted that these records may not be independent but represent, for example, multiple specimens from a hunter’s bag. The probability that there are no sightings between 1980 and 2008 if the species is extant is given by
1- $\hat{\lambda}$*e(t)*) = (1-(*N(T)/I(T)*),

i.e. $\left\{ 1-\left( \frac{no. years in which focal species sighted}{no. years in which any species sighted} \right) \right\}$^ (*total effort in the period (*$\tau,T]$)

Taking the focal species as the king quail, *N(T)* = 3, *I(T)* = 12 and hence $\hat{\lambda}$= 0.25. If *I($\tau$)*is 1980, the total effort in the period ($\tau,T]$*)*is 3 and so *p* = (1-0.25)^3^ = 0.421875. Since *p* < 0.5 we infer that the king quail has become locally extinct in this cell.

Supplementary Material 6. Results for examination of factors affecting the occurrence of ‘data-absent’ cells, i.e. cells containing records dating from pre- but not post-1980.

| Realm | Covariate | | | | | N | AIC |
| --- | --- | --- | --- | --- | --- | --- | --- |
|  | Intercept | PA (TRUE) | Forest | Grassland | Mean HII |  |  |
| Palearctic | -2.080 (±0.106)*** | 0.784 (±0.066)*** | 0.211 (±0.071)** | -0.080 (±0.112) | 0.108 (±0.004)*** | 5945 | 6574.5 |
| Indo-Malaya | 0.071 (±0.207) | 1.167 (±0.099)*** | -0.128 (±0.116) | -0.841 (±0.142)*** | -0.032 (±0.008)*** | 1985 | 2558.1 |

Supplementary Material 7. Model selection results for response variables evaluating the percentage of data-absent cells per country. All models include an intercept plus additive terms shown. K is the number of parameters estimated in the model, ΔAIC_c_ is the IC distance of a model from the minimum AIC_c_ model, *w_i_* is the AIC_c_ weight of a model. We present only those models with ΔAIC_c_ < 2.

| Model | Effect Size | | | Log-likelihood | K | AIC_c_ | ΔAIC_c_ | *w_i_* |
| --- | --- | --- | --- | --- | --- | --- | --- | --- |
|  | GPI | English_Lang | Log(GDP) |  |  |  |  |  |
| GPI | 0.310 (± 0.067) *** |  |  | -434.20 | 3 | 874.69 | 0.00 | 0.44 |
| GPI + English_Lang | 0.327 (± 0.068) *** | -13.8 (±10.5) |  | -433.33 | 4 | 875.13 | 0.45 | 0.35 |
| GPI + log(GDP) | 0.253 (± 0.094)** |  | -2.86 (± 3.29) | -433.81 | 4 | 876.10 | 1.41 | 0.22 |

Supplementary Material 8. Species richness counts per cell under the different assumptions.

| No. Species | No. cells | | | |
| --- | --- | --- | --- | --- |
|  | Assumption (i) | Assumption (ii) | Assumption (iii) | Assumption (iv) |
| 1 | 3448 | 1485 | 1820 | 1922 |
| 2 | 2170 | 1430 | 1379 | 1425 |
| 3 | 1245 | 938 | 876 | 820 |
| 4 | 661 | 493 | 427 | 356 |
| 5 | 468 | 320 | 285 | 245 |
| 6 | 267 | 216 | 179 | 187 |
| 7 | 168 | 121 | 97 | 88 |
| 8 | 89 | 72 | 56 | 50 |
| 9 | 48 | 33 | 23 | 27 |
| 10 | 38 | 30 | 16 | 20 |
| 11 | 29 | 15 | 9 | 7 |
| 12 | 24 | 12 | 9 | 6 |
| 13 | 8 | 9 | 4 | 0 |
| 14 | 5 | 4 | 3 | 1 |
| 15 | 4 | 1 | 1 | 0 |
| 16 | 3 | 0 | 0 | 0 |
| 17 | 2 | 0 | 0 | 0 |

Supplementary Material 9. The difference in number of species estimated as present per cell post-1980 between the most conservative assumption (i) that no local extinctions have occurred) and the least conservative assumption (iv) that all data-absent cells post-1980 represent local extinctions. Dark blue 1-2 species; light blue 3- 4 species; yellow 5-7 species; orange 8-11 species, red 12-17 species.


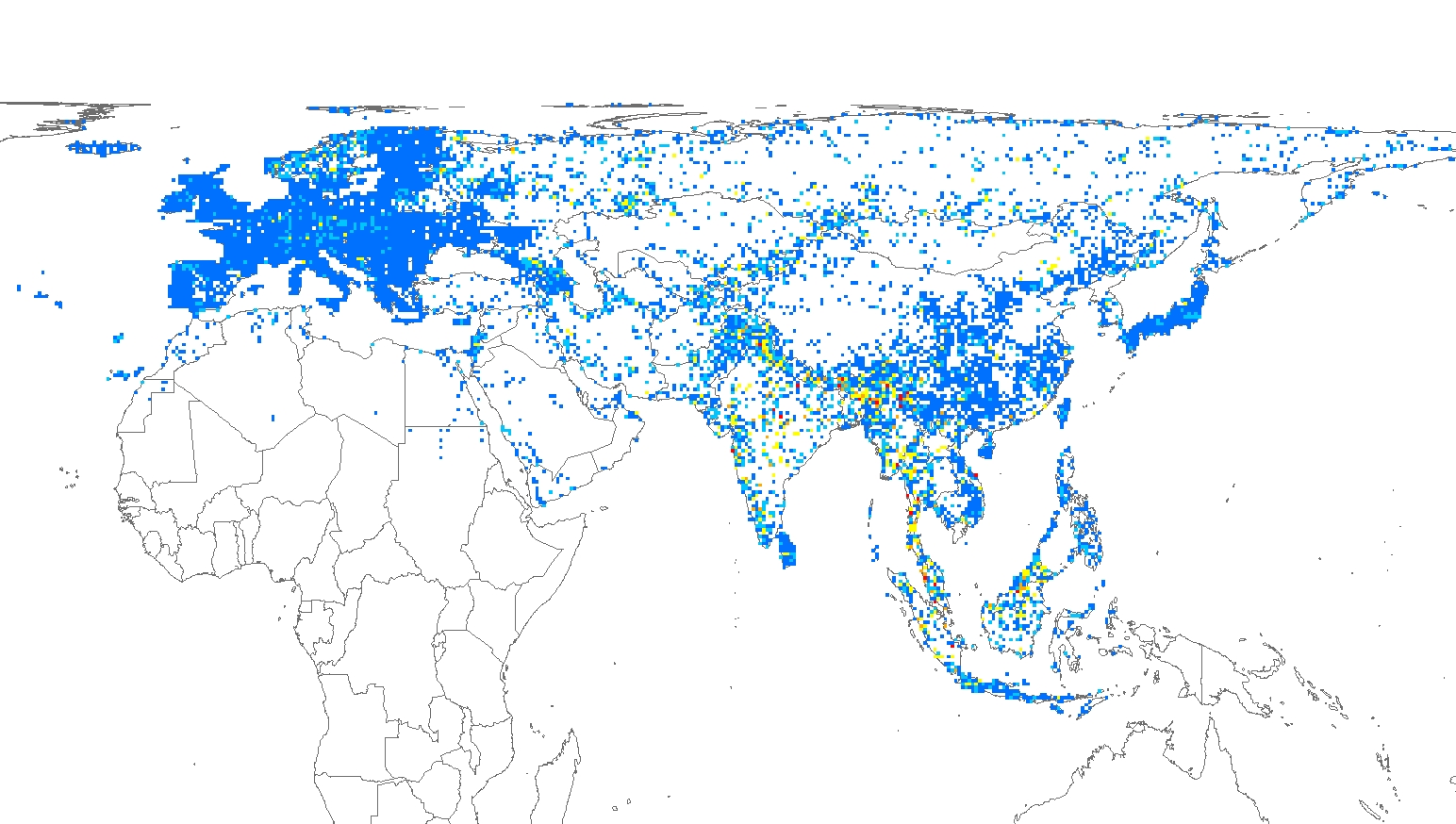


Supplementary Material 10. The EOO polygons of the 21 species for which areas were <50% of the most conservative assumption (i) (in black) as calculated using assumption (ii) (in red).


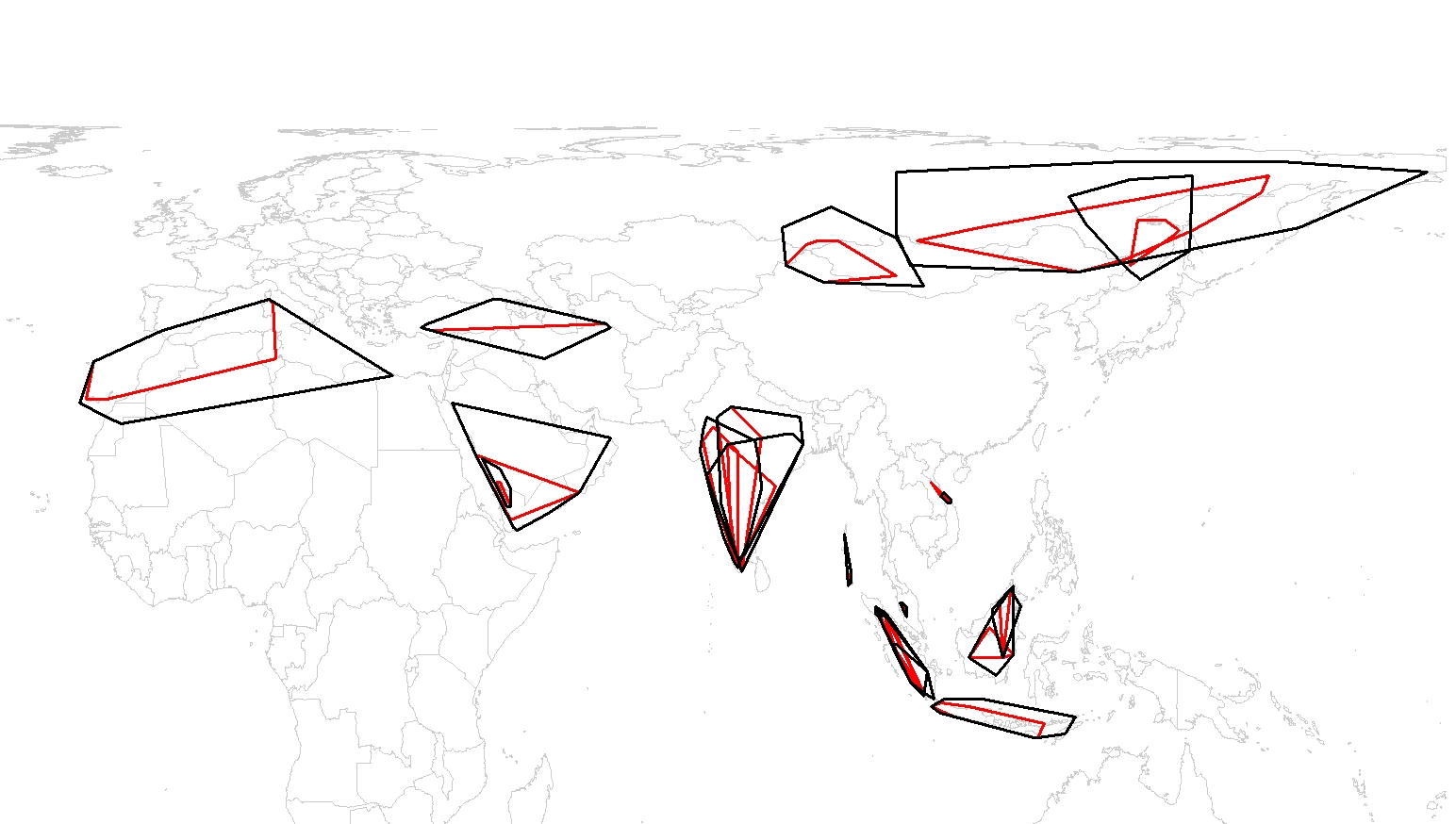

Supplement: BoakesEtAlBiolLettersSupplMaterial3-10 [file rsbl20150824supp3.docx]
